# 

**Published:** 1864-10

**Authors:** 


### CONTENTS.

### ORIGINAL CONTRIBUTIONS.

ART. XXXIV.—OBSERVATIONS ON PUERPERAL FEVER. By DELASKIE MILLER, M.D., Chicago, ........ 529
ART. XXXV. CASE OF HEPATIC DISEASE. By D. B. TRIMBLE, M.D., Chicago, ........ 538
ART. XXXVI. REPORT OF THE COMMITTEE ON SURGERY. By E. ANDREWS, M.D.. Prof. of Surgery in Chicago Medical College, ........ 541

### THE CLINIQUE.

MEDICAL WARDS OF THE MERCY HOSPITAL. CHRONIC DIARRHŒA—CAMP DIARRHŒA, &c. By N. S. DAVIS, M.D., Prof. of Practical and Clinical Medicine, ........ 561

### PROCEEDINGS OF SOCIETIES.

Fox River Valley Medical Association, ........ 565

### SELECTIONS.

Pathology and Treatment of Asthma. By Hyde Salter, M.D., F.R.S., &c. On the Treatment of Asthma by the Iodide of Potassium, ........ 570
Dietary in Disease. By Edward Smith, M.D., &c., ........ 457
On the Hypodermic Treatment of Uterine Pain. By J. Henry Bennet, M.D., late Physician-Accoucher to the Royal Free Hospital, ........ 580

### BOOK NOTICES.

A Treatise on Gonorrhœa and Syphilis. By Silas Durkee, M.D., ........ 583
Outlines of Surgical Diagnosis. By George H. B. Macleod, M.D., F.R.C.S.E.; &c., ........ 584
Military, Medical, and Surgical Essays. Edited by Wm. A. Hammond, M.D., Surgeon-General U.S.A., ........ 584
A Systen of Surgery; Pathological, Diagnostic, Therapeutic, and Operative. By Samuel D. Gross, M.D., ........ 585

### EDITORIAL.

Chicago Medical Society, ........ 586
Chicago Medical College, ........ 586
A New Ambulance, ........ 587
Promotion, ........ 587
Surgeon-General of U.S.A., ........ 587
Explanation, ........ 587
Symptoms of Poisoning Following a Dose of Disulpate of Quinine, ........ 587
Uterine Action During Sleep, ........ 588
In the Vomiting of Pregnancy, ........ 588
A Forearm Wrenched off during Efforts at Reduction for Dislocation, ........ 589
Paraplegia, ........ 589

